# Present Status in Polymeric Mouthguards. A Future Area for Additive Manufacturing?

**DOI:** 10.3390/polym12071490

**Published:** 2020-07-03

**Authors:** Ana M. Sousa, Ana C. Pinho, Ana Messias, Ana P. Piedade

**Affiliations:** 1 Department of Mechanical Engineering, University of Coimbra, CEMMPRE, 3030-788 Coimbra, Portugal; amicaelabsousa96@gmail.com (A.M.S.); acspinho5@gmail.com (A.C.P.); 2 Faculty of Medicine, Dentistry Department, University of Coimbra, CEMMPRE, 3000-075 Coimbra, Portugal; ana.messias@uc.pt

**Keywords:** polymeric mouthguards, orofacial injuries, polymers, processing, 3D printing

## Abstract

Athletes from contact sports are more prone to orofacial injuries because of the exposure to possible shocks and collisions derived from physical proximity. The use of protector polymeric mouthguards proved to be useful in the prevention of the described injuries. There are different types of mouthguards with varying ranges of protection and prices, but they are all made from polymers and share the same propose: to absorb and dissipate the impact energy resulting from the shocks. As they are used inside the mouth, they should not impair breathing and speaking nor compromise the comfort of the athlete. However, the ideal mouthguard is yet to be created. The choice of the most appropriate polymeric material and the standard required properties have not yet been reported. Regardless of the numerous studies in this field, normalized control parameters for both material characterization and mouthguard fabrication are absent. This paper aims to present a review of the current types of available mouthguards and their properties/characteristics. Moreover, a detailed description of the most common polymers for the fabrication of mouthguards, together with the manufacturing techniques, are discussed.

## 1. Introduction

Orofacial injuries are a class of trauma that includes tooth fracture, laceration or luxation of soft tissues (tongue and gums), facial bone traumas, and damage to the temporomandibular joint [1,2]. It is estimated that a quarter of the US population aged 5–60 years suffers from a particular dental trauma throughout their life [3]. Unfortunately, in some cases, the treatment for such conditions is quite expensive or can lead to permanent damage [4]. 

An increase in orofacial injuries during training and competition has been observed in several sports. Herein, athletes are frequently exposed to severe blows and impact shocks that result from physical contact [5]. These injuries represent around 18% of all sports injuries, and more than half of them are associated with dental trauma [6]. As expected, the highest orofacial trauma prevalence is related to contact sports. Conducted surveys helped to estimate the approximate percentage of practitioners that experienced orofacial injuries of any type: wrestling (83.3%), boxing (73.7%), basketball (70.6%), karate (60.0%), jiu-jitsu (41.2%), handball (37.1%), football (23.3%), judo (22.3%), and field hockey (11.5%) [7,8]. 

In an attempt to mitigate the numbers, in 1950, the American Dental Association (ADA) suggested using protective devices known as mouthguards [9]. A decade later, after proving their effectiveness in the prevention and attenuation of orofacial injuries, the use of mouthguards became mandatory for physical contact [10]. Nowadays, ADA recommends using protective mouthguards for 29 different activities and sports, including all martial arts, wrestling, boxing, ice and field hockey, American football, basketball, handball, water polo, volleyball, and volleyball in-line skating, and gymnastics, among others [11,12]. 

Mouthguards are described by the American Society for the Testing of Materials (ASTM) as “a resilient device or appliance placed inside the mouth (or inside and outside), to reduce mouth injuries, particularly to teeth and surrounding structures” [13]. Briefly, a mouthguard’s primary function is to absorb, dissipate, and distribute the forces resulting from shocks during physical activity [9,14]. Nonetheless, the device should not jeopardize the athlete’s performance by impairing the ability to breathe and communicate [15]. 

The present paper intends to present a guidance review of the existing types of mouthguards. Also, special attention is given to the base polymers used for the fabrication of these devices. The manufacturing processes and techniques are also discussed.

## 2. Properties and Design of Mouthguards

Regardless of the type of mouthguard available in the market, the device must fulfill some requirements to provide both protection and comfort, at an affordable cost [16,17]. 

First, the devices must be made from tasteless and non-toxic polymers. Additionally, they should present high impact resistance to achieve proper absorption and redistribution of the shock energy over a large area, and consequently, reduce the probability of injury [9,18]. Other properties, such as water absorption, stiffness, hardness, and material processing should also be taken into consideration to match the desired performance of the device [19].

The protectors inside the mouth must be aligned with the upper jaw. Therefore, a proper fit and high local retention throughout the physical activity constitute the design challenges to consider for the device to remain in the same position during the sports practice [20]. It is also noteworthy that the thickness through the length of the device may affect the final retention, comfort, and capacity of protection [21]. Therefore, to achieve an ideal design, it is necessary to know the extent of the tissue that needs to be protected, to identify the limits, the outline design, and the thickness of the mouthpiece in each surface [22].

**Figure 1:**
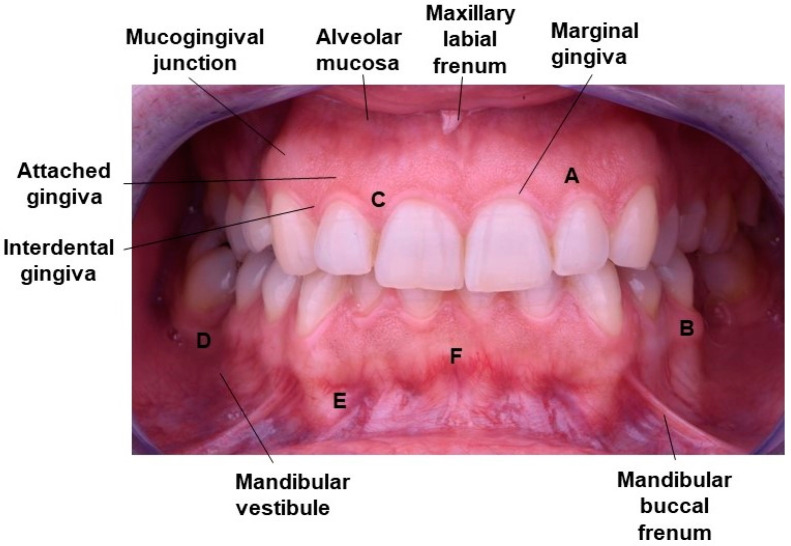
Clinical appearance of gingiva: (**A**) Attached gingiva above and interdental papilla below; (**B**) mucogingival line separating attached gingival from mucosa; (**C**) free gingival margin; (**D**) posterior vestibular fornix; (**E**) anterior vestibular fornix or mucobuccal fold; (**F**) frenum area.

The manufacture of mouthguards with accurate borders implies that the region of gingiva margin and vestibular area (vestibule) should be considered in the design (Figure 1) [23]. The vestibule also includes the flanges which are named according to the surface covered by the vestibule (labial, buccal, and lingual) [24,25]. 

**Figure 2:**
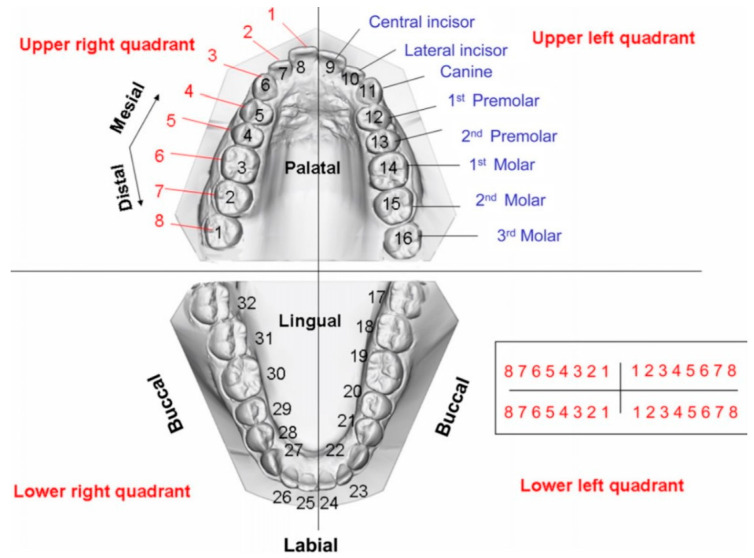
Maxillary and mandibular dental arches. The individual teeth can be numbered sequentially from mesial to distal in each quadrant (indicated in red) or uniquely numbered, starting with the right maxillary third molar as tooth number 1 (shown in black). The orientation of the teeth is indicated by labial, buccal, lingual, and palatal as reference. The relationship between teeth is indicated by mesial-distal terms [26]. Published by Elsevier.

Furthermore, mouthguards must protect the hard tissues of the mouth, including teeth and surrounding structures. All teeth and tooth surfaces of the upper maxillary dental arch are identified in Figure 2.

The process of the ideal mouthguard starts by taking an alginate impression of the mouth. Then, a calestone stone powder is poured into this alginate impression under vibration, to ensure that the calestone mixture reaches the most profound details, and is left to dry and settle into a stone-like model [27]. After this step, border imperfections are carefully eliminated. The stone model must include all the teeth until the second molars. More specifically, it should consist of the distal surface of these teeth, the palatal or lingual surface, the labial frenum, and buccal margins.

**Figure 3:**
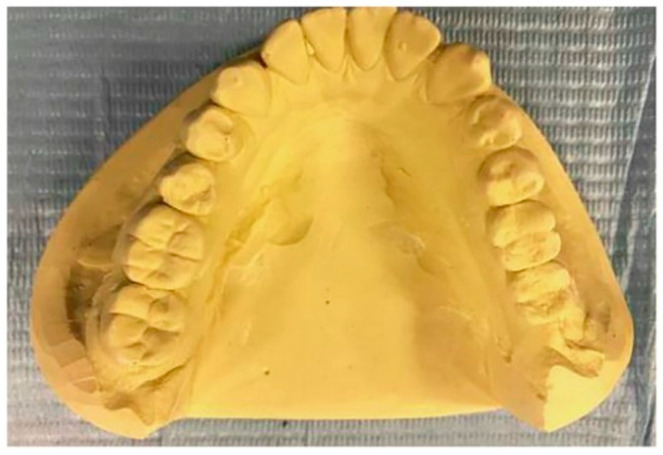
Example of a dental stone model [31]. Published by Elsevier.

Furthermore, the dental model cast should be long enough to cover until the vestibular area, especially the labial flange in the range of 2 mm. The limit of the labial flange must be curved while the palatal border should be tapered [28,29]. The tooth cusps, which are defined as “*pyramidal elevations located on the occlusal surfaces of the molars, premolars, and the incisal edges of canines*” should also be included in the mold [30]. A dental stone model to manufacture a mouthguard is displayed in Figure 3.

**Figure 4:**
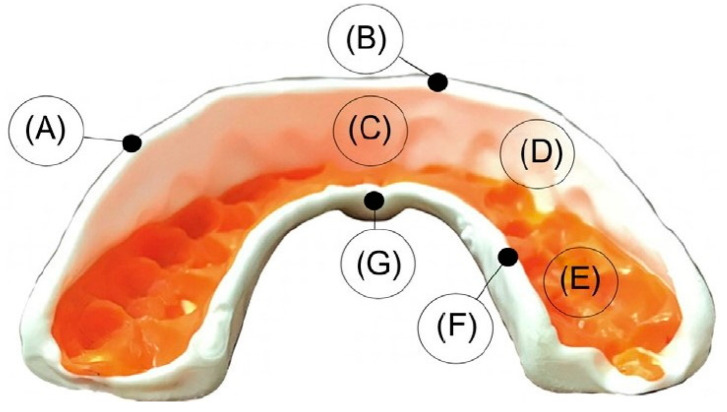
Basic design of a mouthguard: (**A**) edge of buccal flange; (**B**) edge of labial flange; (**C**) labial flange; (**D**) buccal flange; (**E**) occlusal surface; (**F**) edge of palatal flange; and (**G**) palatal flange [38]. Published by Elsevier.

As aforementioned, the thickness across the protector is of extreme importance. According to the literature, the incisal edges and tooth cusps are considered critical areas of the mouthguard and must be nearly 4-mm thick [32]. Additionally, the region of mouthguard that includes the labial surface must tolerate a thickness of 3 mm. An insufficient and/or irregular thickness on the occlusal surface may lead to deformation in the jaw and consequent fracture. To prevent these problems, the device should have a thickness of 2 mm in this region, allowing a stable occlusal contact [33,34,35]. Finally, the palatal surface must have a thickness of approximately 1 mm, while the palatal flange should have an extension of 10 mm over the gingival margin [36,37]. A basic design of mouthguards, including all the required surfaces, is shown in Figure 4.

Despite the extent of studies regarding the thickness of a mouthguard, some studies report that the thickness control parameters remain non-rigorous and variable, leading to the production of protectors with inadequate profiles that provide decreased protection capability [39].

## 3. Types of Mouthguards

The present section aims to provide a detailed description of the different types of mouthguards reported in the literature while discussing the main advantages and limitations of their use.

The manufacturing and design of these devices are regulated by the ASTM F697-16 standard, entitled “Standard Practice for Care and Use of Athletic Mouth Protectors” [13]. According to this standard, there are three main types of mouth protectors with respective classes, as shown in Table 1.

However, both in literature and sports environment, such devices are commonly classified into three different designations: stock, “boil and bite,” and custom-made mouthguard [40]. These categories arise from the molding technique and design associated with each mouthpiece, facilitating identification and recognition. For this reason, in the present manuscript, the description of mouthguards is performed according to the most common terms used in the literature to favor comprehension and further research. All types share design features that match the anatomical area of the mouth where they are used.

### 3.1. Stock Mouthguard

**Figure 5:**
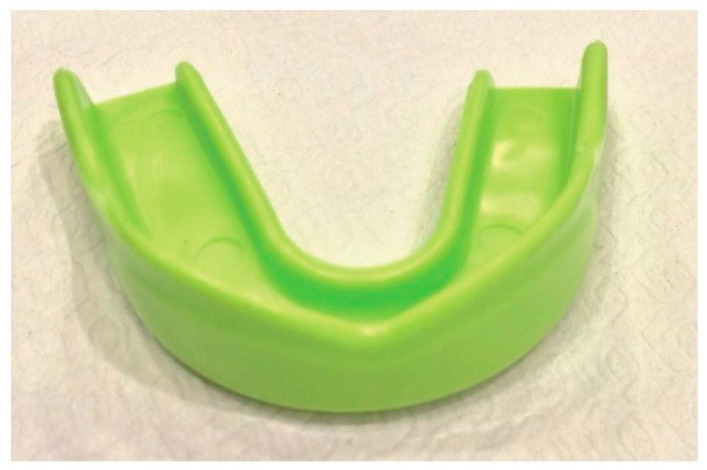
Stock type mouthguard [41].

Stock mouthguards present the most straightforward design, are prefabricated and available in different sizes, allowing the athlete to choose the protector that offers the better fit to its dental arch [28]. One example of a stock mouthguard is displayed in Figure 5.

The main advantage of this mouthpiece is that it is commercially related, as they are available in the majority of sports stores at the lowest prices, accessible to a broader range of athletes. However, stock type mouthguards have several drawbacks, especially concerning their fit [36]. Because of the incapability to adjust fully to the mouth, constant repositioning may occur, leading to possible loss of the device during physical activity. The misfit directly affects the ability to absorb and dissipate energy resulting from impact forces [42]. Another disadvantage is associated with the discomfort provided to the users. In a study led by Queiróz et al. all athletes involved stated that it is impossible to communicate with their colleagues while wearing this protector [43]. Besides, symptoms of nausea and difficulty in breathing have also been described. Therefore, specialists often advise athletes not to use this type of mouth protector, unless they are undergoing orthodontic treatments, or as an emergency in case of loss or destruction of a more appropriate device [44,45].

### 3.2. Mouth-Formed Mouthguard

**Figure 6:**
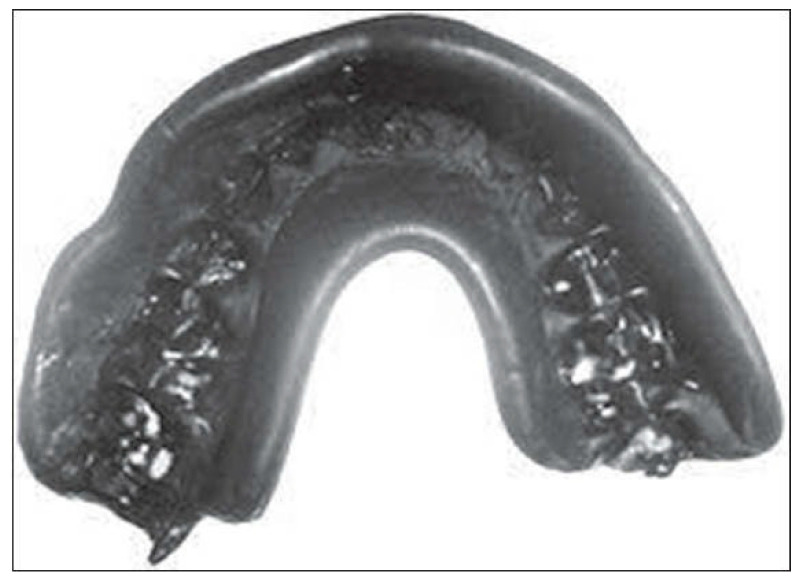
A mouth-formed mouthguard (“boil and bite” mouthguard) [47].

The mouth-formed mouthguards are commonly known as “boil and bite,” which derives its name from its molding process [37]. These devices are prefabricated from thermoplastic polymers that enable their molding and consequent dental arch imprinting through heating. Regardless of the difficulty of producing a satisfactory dental impression, as pointed out by dentistry specialists, the molding process, associated to these protectors, is simple and does not require any dentistry-related data [45]. The molding procedure begins by immersing the mouthpiece in hot water until the polymer becomes malleable (above glass transition temperature). Then, the user places the protector into the mouth and lines up the device with the centerline of the upper teeth. The mouthguard is then pressed against all the teeth and gum of the upper jaw. To conclude the procedure, the user hardly bites down the structure to print the shape of the soft and hard tissues of the mouth into the mouthguard [46] (Figure 6).

The quick molding procedure, the affordable price, and the accessibility-related with “boil and bite” protectors increased their popularity and use among the sports community representing nearly 90% of the mouth protectors used by athletes [37]. It should be noted that they provide an enhanced degree of protection compared with the stock type devices. Nonetheless, some authors such as Duddy and his colleagues demonstrated that athletes still experience difficulty in breathing and speaking, which might impair their performance during the sports activity [48,49]. Moreover, Queiroz et al. stated that 80% of the evaluated athletes studied could not communicate [43]. Another disadvantage concerning the use of a “boil and bite” protector is associated with the processing methodology. Studies proved that, if the device is bitten with enough force, the occlusal thickness can decrease substantially and compromise the protection provided by the mouthpiece [36]. According to Park and co-workers, the protector’s thickness can decrease around 70–99% after the conformation procedure, because of extreme or uncontrolled pressure applied during the biting step [50]. These data are complemented by other research that mentions that the device might suffer high deformation when exposed to the pressure of the teeth since it is manufactured using a thermoplastic polymer [51]. Thus, it is possible to conclude that a significant thickness decrease occurs when the mouthpiece is used for a certain period, compromising the ability to provide proper protection. 

### 3.3. Custom-Made Mouthguard

Because of the limitations associated with the previously described protectors, there was the need to create a device with enhanced fit and accommodation to the dental arch. The custom-made mouthguards were designed to overcome these limitations. Instead of being prefabricated, health professionals manufacture these protectors through a personalized mold of an individual mouth, in specialized laboratories [30]. Because of the correct adjustment, the impact resistance and shock absorption are enhanced without compromising the comfort of the athlete [52].

**Figure 7:**
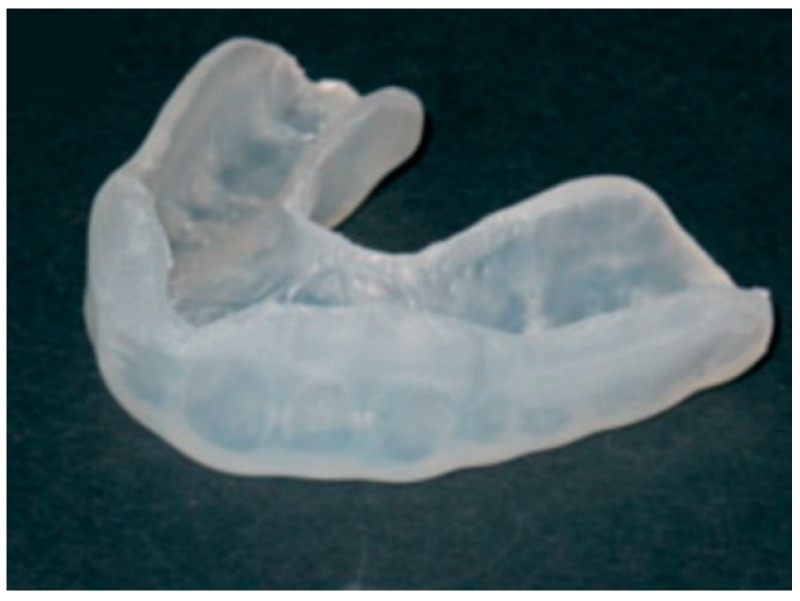
A custom-made mouthguard for the upper jaw ex-situ (adapted) [48]. Copyright Wiley-VCH Verlag GmbH & Co. KGaA. Reproduced with permission.

The manufacturing process of these devices is based on a dental arch scan printed on a stone-like model [22]. After this step, this final model is subjected to a thermoforming procedure in which a thermal moldable material covers it. A maximum adaptation to the mouth of the sportiest is achieved as a result of a well-fitted model. Furthermore, the properties of custom-made mouthguards may differ depending on the thermoforming process used, as described later in the present manuscript. However, this process presents the disadvantage of the cost associated with the mold production. Each mold is only suitable for one specific user to ensure the perfect fit. One example of a custom-made device is displayed in Figure 7.

According to the literature, these protectors can be further classified into single-layer type and laminated-type. As the designation suggests, the first ones are created with one single layer. On the other hand, the laminated-type is constituted by more than one layer of thermoplastic sheets fused tightly. Laminated mouthguards provide better stability than single-layered because of lower stress accumulation during the fabrication process [53]. Unlike the single-layer type, laminated mouthguards can maintain the appropriate thickness that guarantees higher shock absorption capacity [54].

Notwithstanding, the laminated-type also has some drawbacks. Over time, these protectors show a tendency to delaminate. The weak interfacial adhesion can explain this fact, which may be related to contamination of the polymeric sheets’ surface and the temperature of the thermoforming process. Higher temperatures usually imply stronger bonds [55,56,57]. 

Several authors have stated that custom made mouthguards are the most effective as they provide higher retention, protection, comfort, and do not impair the performance during the physical activity [58,59]. It was proved that, in different sports modalities, custom-made devices produced better results in tests related to the athletes’ breathing performance in comparison to other types of protectors. The users did not reveal discomfort or any difficulty in speaking [43,60]. Recently, hockey athletes manifested their preference for custom protectors during training and competition, based on their comfort [61]. 

Despite the improved adaptability to the athletes’ features, these devices have a few associated disadvantages. First, the protector is manufactured only by pre-order, which increases its market value, especially for the laminated-type [45,62]. Another drawback is related to the fabrication technique that may lead to insufficient thickness along the device, causing variations of the properties of the custom-made mouthguards [46,50]. These changes occur because of the lack of effective quality control in the manufacturing process [39]. 

Another fact that must be highlighted is the difficulty in establishing a direct comparison of the performance of the custom-made mouthguards reported in the literature. This happens because of the lack of standard guidelines for material testing. An example is related to the evaluation of the energy absorbed by the protector. It is quite common to find diverse methodologies using different evaluation methods and impactor instruments. In some cases, even information about the size of the testing probes is missing [17,42]. Consequently, it would be of extreme importance to set testing guidelines and performance requirements to assist new research in this specific area.

## 4. Mouthguard Base Material

Polymers are, unquestionably, the appropriate class of materials for the production of mouthguards. Polymers have the flexibility to be chemically modified or serve as the matrix of several types of nanocomposites with specific surface properties [63,64,65]. For the production of mouthguards, natural rubber, silicone, polyvinyl chloride, polyurethanes, or polyolefins have been used. The following section presents a description of the most commonly used materials for the production of the protective devices while discussing their properties and benefits for this particular application.

### 4.1. Ethylene Vinyl Acetate (EVA) Copolymer

**Figure 8:**
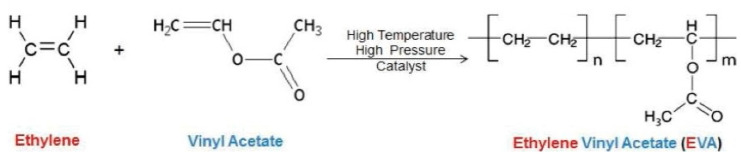
Synthesis scheme of the synthesis of ethylene vinyl acetate (EVA) copolymer [72].

Currently, ethylene vinyl acetate (EVA) copolymer is the most common polymer used for the preparation of mouthguards since it can be adapted to the design of any type of device [37,40,66]. EVA is a thermoplastic copolymer, easily manipulated when heated. Additionally, it is considered non-toxic and easily available in the market [67,68]. This copolymer synthesis is conducted by high-pressure free radical polymerization of ethylene and vinyl acetate (VA) monomers resulting in a random copolymer [69,70,71]. A scheme of the EVA synthesis is shown in Figure 8.

The behavior of the final copolymer depends on the proportion ratio of both ethylene and VA units. The higher the VA content, the lower the percentage of crystallinity of the final material [70]. For VA ratios higher than 50%, the material is amorphous and transparent [70]. Additionally, the VA ratio affects the melting temperature (*T*_m_), as demonstrated in a study conducted by Arsac et al. where the Tm increased from 55.5 °C to 104.5 °C for a VA content of 40% and 5%, respectively [73]. Similarly, an increase in the VA content results in the enhancement of other features like the solvent solubility, flexibility, material clarity, and toughness [38]. The physical properties of EVA can also be tuned by adding pigments or varying the average molecular weight through copolymerization with other monomers [74]. A study performed by Del Rossi and co-workers showed that adding a pigment to the EVA copolymer changed the material properties. Herein, the dark colors present better retention and fit than the transparent material as they absorb more energy [75]. Additionally, EVA structure can be degraded by several physical factors, like UV radiation, gamma radiation, and temperature [76]. 

This thermoplastic material has interesting properties, such as its impact and corrosion resistance. Compared to other polyethylene resins, EVA is more flexible and very similar to rubber [68]. Moreover, this copolymer presents good adhesion to an extensive range of materials, like ceramics, metals, and other polymers [76,77]. 

Most sports mouthguards are manufactured from EVA with a 28% ratio of VA. This percentage allows favorable properties for this application, such as high impact energy absorption, which is increased when exposed to body temperature [19,78].

Some EVA-based mouthguards can include air insertions or other polymeric materials like polyamides, poly(ethylene terephthalate glycol) (PETG), or polyurethanes (TPU) to improve specific properties [19,79]. Westerman and his colleagues showed that EVA mouthguards with air inclusions display better energy absorption, and consequently, less transmitted forces to the teeth and surrounding structures [68,80]. The same authors also tested the possibility of adding hard inserts into EVA mouthguards [81]. Herein, it was verified that the EVA mouthguards with the EVA hard inserts presented lower absorption of impact energy when compared with simple EVA mouthguards. Besides, the position of these hard inserts influenced the ability to absorb impact energy. When they are closer to the impact surface, the energy absorption decreases [81]. The combination of three approaches was tested in a study conducted by Takeda et al. [82]. This work compared the buffer capacity of three different EVA mouthguards where the first was a simple laminated device, the second had hard inserts of acrylic resin, and the last one combined the acrylic hard inserts with air inclusions. The results showed that the EVA laminated type from the three devices presented the lowest performance concerning the shock absorption. On the other hand, the third type (acrylic resin hard inserts plus air inclusions) delivered the highest shock absorption performance [82]. 

Regardless of the frequent use of EVA in mouthguards, some drawbacks can also be pointed. First, in comparison with other materials, EVA has a higher swelling capacity, which implies that it will absorb more water from the oral cavity, increasing the probability of dimension variations, and consequently, causing discomfort to the athlete [83]. Also, lower stiffness leads to lower energy dissipation and deficient tension distribution. A study conducted by Cummins et al. proved that EVA has low stiffness (9 MPa) and cannot achieve load redistribution, even when the thickness increases [84]. Because of these and other disadvantages, some questions arose concerning the choice of EVA as the polymeric base material for mouthguards. Further studies need to be conducted to fully understand if EVA is the appropriate material for sports mouthguards since there are no standardized methods to evaluate the dissipation and absorption energy of EVA when using different testing equipment and test specimens.

### 4.2. Polyolefins

An olefin, also known as an alkene, is an unsaturated molecule containing a minimum of one carbon double bond [85,86]. Olefin monomers are produced through the cracking of crude oil, which is also named steam cracking [87]. The most produced polyolefins are polyethylene (PE), polypropylene (PP), and polybutylene (PB) [88]. 

In general, polyolefins are quite versatile and easy to process and recycle. These facts associated with their low price increased their use in a wide range of applications [89]. The majority of polyolefins has high chemical resistance and low melting temperature [88,90]. The mechanical properties of these polymers can be fine-tuned through blending with other polymers, additives, or by copolymerization [91].

In dentistry, the first reported application of these polymers was for the production of dentures. With the increased knowledge of these materials’ properties, they started to be pointed as good alternatives for custom-made mouthguards production [57,92]. Compared with EVA, these polymers show higher tensile and tear strength with similar shock absorption capacity [92]. Additionally, polyolefins absorb less water from the oral cavity, which may prevent unwanted swelling, dimensional variations, or bacterial contamination of the protector [93,94]. In the case of mouthguards constituted by laminated sheets, polyolefins showed better interlayer adhesion than EVA sheets, which is essential to prevent the delamination of the device [66]. Polyolefin-based devices also present better outcomes in preserving the thickness of the mouthpiece after the thermoforming process than EVA-based ones [95].

Despite the apparent better performance of polyolefin-based mouthguards, further studies establishing the comparison between the two materials, in trials with athletes, are still lacking in the literature. For example, it is also crucial to understand if the comfort and performance of the athletes are compromised when used on a daily basis.

### 4.3. Other Materials

Several materials have been pointed out as substitutes of EVA for the production of mouthguards. Polymeric materials, such as polyvinyl chloride (PVC), polyurethanes, or acrylic resins, are examples that have been cited since 1970 [96]. Nevertheless, some of them were excluded because of potential toxicity, which is the case of PVC [36]. 

Thermoplastic polyurethanes (TPU) are frequently used as the base material for mouthguards. These elastomeric polymers are easy to process, and like EVA, present high shock absorption making them good candidates for this application [74,97,98]. Additionally, they can be intercalated with EVA layers, enhancing the energy absorption capacity of the protector [19,99]. Regardless of these good indicators, studies, where the athletes use a TPU-based mouthguard, have not been reported.

Other polymers such as silicone rubbers are used for mouth protectors [96,97] because of their shock absorption capacity [100,101]. Auroy and colleagues proved that room temperature vulcanized silicone rubbers (RTV) have a better shock absorption capacity than EVA [102,103]. Once again, there are no studies that evaluated the retention and fit of a silicone rubber-based mouthguard in athletes, indicating the need to increase the knowledge of the performance of these polymers in this specific application. 

Using a different approach, McNair and co-workers tested the possibility to prepare a mouthguard using thermosetting polymers based on ternary thiolene systems modified with urethane (UMTEN) or acrylate (AMTEN). Compared with EVA, they presented higher water absorption, which may affect the dimensional integrity of the device and compromise the comfort of the users [51]. 

Some researchers produced mouthguards by overlapping two sheets of different material, TPU, and PETG, with a final thickness of 3 mm [104]. Their objective was to study the effect of sports drinks jointly with mouth protectors in the increase of caries. They found that wearing a mouthguard after consuming sports drinks is a risk factor for dental caries.

More recently, new approaches have been described employing technical engineering polymers like polyetheretherketone (PEEK). The tests showed that PEEK is not suitable for mouthguards as they presented a reduction in the thickness of the occlusal surface, after the forming process, compromising the device protection capacity [105].

## 5. Fabrication Techniques of Custom-Made Mouthguards

Despite the chosen technique, the manufacturing process of a custom-made mouthguard follows a series of stages, including the preparation of the mouth prototype, the manipulation of the selected thermoplastic sheet, and the final forming procedure. All steps are conditioning since they have a direct influence on the thickness and final precision fit of the mouthguard [55]. These factors are critical to minimize the impact of physical activity and provide the maximum comfort to the athlete.

The manufacturing process of custom-made mouthguards generally induces a variation in the thickness of the original sheet material because of the forming process, heating temperature, and polymer thickness and shape, among others [106,107]. The used polymeric sheets are available in the market in several dimensions, shapes, and colors to further personalize the protector. The thicknesses range from 1 to 4 mm, the latest being most used for the fabrication of single-layered mouthguards [108].

For EVA sheets subjected to the thermoforming processes, it is reported that the ideal temperature range is between 80–120 °C. For polyolefins, on the other hand, the optimal temperature is in the range of 105 to 230 °C [94,108]. After the forming step, the sheet cooling process should also be carefully optimized to prevent the distortion of the final device [21]. 

Different forming processes and equipment can be used to manufacture custom-made protectors, the most common the pressure-forming, the vacuum-forming, or a combination of both methods [95,109]. The following section presents a brief description of the fabrication methods of custom-made mouthguards reported in the literature.

### 5.1. Pressure-Forming Technique

Although there are different types of pressure apparatus, all share the same working principle in which they pull a heated sheet over a mouth mold. Usually, pressure forming sets are equipped with a circular holder, which allows the use of rounded-shape polymeric layers [110]. Besides the holder, where the sheet is engaged, the apparatus also has a heating and a pressure unit. The heating unit is placed above the sample holder while the pressure one is sealed to the holder on the opposite side. The fabrication procedure begins by placing the polymeric sheet into the holder. Then, the heating unit is turned on, and when the desired forming temperature is achieved, the pressure unit is moved onto the mouth mold and is locked in this position. The desired pressure is applied to proceed with the molding of the sheet against the working model for a specific time. After the cooling step, the mouthguard can be trimmed, or the entire process is repeated to add more layers [45].

**Figure 9:**
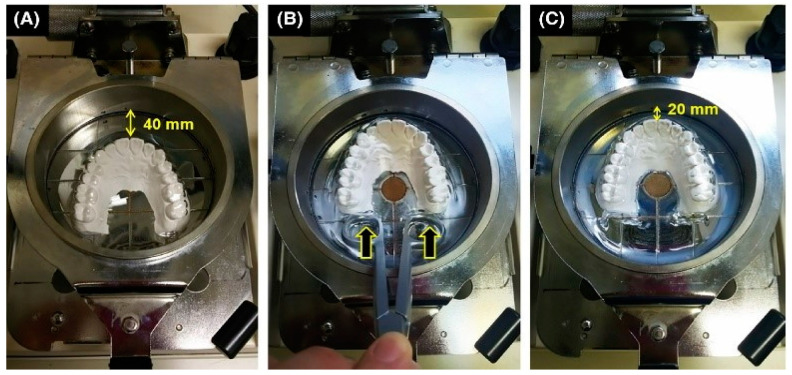
Pressure machine and molding condition (condition MP). The sheet frame at the top of the post was lowered, and the sheet covered the model when it sagged by 15 mm. The rear side of the model was pushed to move the model forward 20 mm, and then, the sheet was formed [110]. Copyright Wiley-VCH Verlag GmbH & Co. KGaA. Reproduced with permission.

Some authors state that the molding conditions in pressure forming generate thinner single-layered mouthguards compared to the vacuum-forming process [35]. Other researchers found that the mouthguard thickness varied between the central incisors and first molars regions [111]. In an attempt to overcome these problems, Takahashi and colleagues proposed a possible solution based on the relocation of the working model, in the forward direction, just before the activation of the pressure (Figure 9) [112].

The pressure forming technique can produce mouthguards with a more precise fit for the working mold compared with the other processing technique [54] despite the thickness reduction.

### 5.2. Vacuum Forming Technique

**Figure 10:**
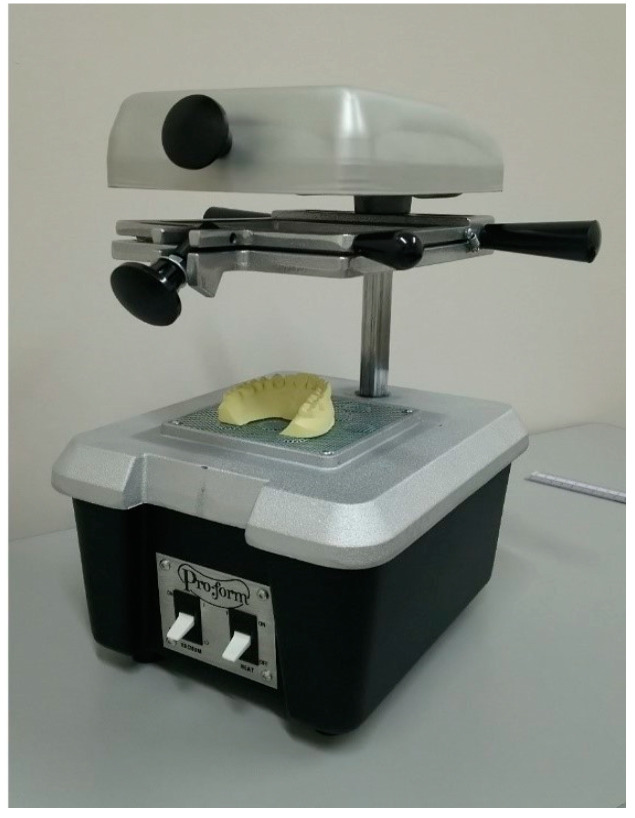
Typical vacuum forming machine (adapted) [54]. Copyright Wiley-VCH Verlag GmbH & Co. KGaA. Reproduced with permission.

In contrast with the pressure forming technique, in the vacuum forming equipment, all elements are displayed vertically, where the vacuum unit is at the lowest level, and the mouth mold is placed on top of it. The sample holder is placed in the middle section, followed by the heating unit, which is placed at the top of the equipment. Similar to the other described technique, the manufacturing process begins by heating the sample, but this step is extended until the sheet sags down. The vacuum unit is then turned on, pulling down the saggy sheet toward the mouth mold. With this procedure, the air is removed from the mold/sheet interface to avoid defects. Finally, the polymeric sheet is cut according to the outline design. Laminated mouthguards are fabricated by sequentially adding a second or third layer [45]. A schematic representation of the vacuum thermoforming process is presented in Figure 10.

As aforementioned, forming techniques may lead to a decrease in sheet thickness. In the case of vacuum forming, this may occur while the material is heated and in the final formation of the mouthguard. Some studies show that with the increase of the heating, the thickness of the mouthguards decreases [108]. Additionally, higher temperatures and longer heating steps increase the sagging reach of the material. For a 4-mm sheet, a proper heating stage is completed when the sag distance is approximately 15 mm [113]. The effectiveness of heat distribution is also significant and is directly related to the shape of the original sheet. Round sheets are associated with thinner mouthguards, which may be a reason for concern [114,115]. Because of the heating unit positioning, the heating temperature should be carefully selected to ensure the correct softening across the sheet and avoid inappropriate fit to the mold [116]. Some types of equipment are equipped with double-sided heating units to correct this problem [108]. 

One advantage of the use of the vacuum formed system is the fact that it induces less reduction of the material thickness when compared with the pressure technique. Mizuhashi et al. proved that a vacuum-formed mouthguard was thicker at the labial surface of the central incisor [35]. However, some studies demonstrate that regardless of the used technique, the thermoforming process itself causes the reduction of the original sheet thickness between 35–60% at the labial surface and 25% at the occlusal surface, which may be insufficient to ensure their protection [95]. Other areas with sharper contours, as the incisal edge or cusp, are also pointed as typical locations where the thickness reduction is evident. On the other hand, smoother areas are formed as the buccal surface suffer from less thickness reduction [30,95].

After the processing, it is necessary to check if the thickness and fit are appropriate. Concerning the final thickness, this parameter is measured in the area of central incisor and first molar at labial, buccal, and occlusal surface. Assessing the fit of the mouthguard is made by measuring the distance between the protector and the cervical margin of the working model [115].

Although thermoforming processes are the most commonly used techniques to manufacture mouthguards, further studies are required to control the production parameters to normalize the mouthguard properties, fit, and thickness.

### 5.3. 3D Printing

More recently, additive fabrication techniques, such as three-dimensional printing (3D printing), have been proposed to manufacture custom-made mouthguards. One of the most significant advantages of 3D printing is producing complex shapes and personalized devices, preferably for a low number of specimens [117,118]. These characteristics correspond entirely to what is desired for the type of device and application considered, where every athlete has a unique anatomic mouth arcade. The acquisition of data to form 3D images and designing and manufacturing devices for dentistry applications are considered as cutting-edge developments in the field [119].

Very few studies are reported in the literature for the fabrication of mouthguards by 3D printing. In a study by Li and co-workers, the use of digital software to design a PEEK-based mouthguard is suggested [105]. They compared the final printed devices with other protectors made by conventional techniques (vacuum-pressure forming), and no significant differences in retention were found. Additionally, the occlusal design of 3D printed mouthguards needed improvement to enhance the impact attenuation. In another study, digital technology was used to create a high-elastic silicone rubber mouthguard proving that this technique can help increase the fit accuracy [120]. However, in this study, the printed material is not suitable for biomedical applications. In a different approach, Liang and colleagues applied the 3D printing technique to prepare oral drug delivery systems in the shape of mouthguards [121]. Despite their shape and positioning, these devices are not intended to provide protection.

Thus, it is suggested that further research is carried out with this technique, applying new polymers, either single or multi-material, to improve the outline design and the thickness of the mouthguard.

## 6. Conclusions

Orofacial injuries are a common disability, especially in the sports environment. The physical contact and lack of protection are the main reasons for this injury incidence. Polymeric mouth protectors, also known as mouthguards, are devices that are created to be placed in the mouth of the athlete during the physical activity, to protect the hard and soft tissues of the mouth. In recent years, the published studies are not focused on the properties of the materials but more on the processing techniques and the testing to understand the efficiency related to the athlete’s comfort and protection. The significant gap is the lack of establishing the proper features and properties that these devices should meet. The only available current standard ASTM F697-16, which classifies mouthguards, uses a terminology that is not the most used or known in both scientific and sports environments. Additionally, the manufacturing process of such devices should also be normalized to set the dimensions and specifications of the protectors. Only then, a proper fit and comfort, would be more easily achieved. 

For future perspectives, the use of digital software and 3D printing are being pointed as alternatives to conventional thermoforming processes used to manufacture protectors. This approach would provide mouthguards with increased thickness precision and design details. Moreover, it can be considered that the use of 3D printing to mouthguards fabrication is an unexploited broad area that can be envisaged as an exciting future area for additive manufacturing.

## Figures and Tables

**Table 1:** Classification of mouthguards according to ASTM F697-16 [13].

Type	Class
I–Thermoplastic type	I–Vacuum-formed
	II–Mouth-formed
II–Thermosetting type	I–Mouth-formed
III–Stock type	--
